# Comparison of the efficiency of TIR1 transgenes to provoke auxin induced LAG-1 degradation in *Caenorhabditis elegans* germline stem cells

**DOI:** 10.17912/micropub.biology.000310

**Published:** 2020-09-18

**Authors:** Jian Chen, Ariz Mohammad, Tim Schedl

**Affiliations:** 1 Department of Genetics, School of Medicine, Washington University in St. Louis, Missouri 63110.

**Figure 1. Comparison of TIR1 transgene mediated degradation in C. elegans germline stem cells by qRT-PCR:**
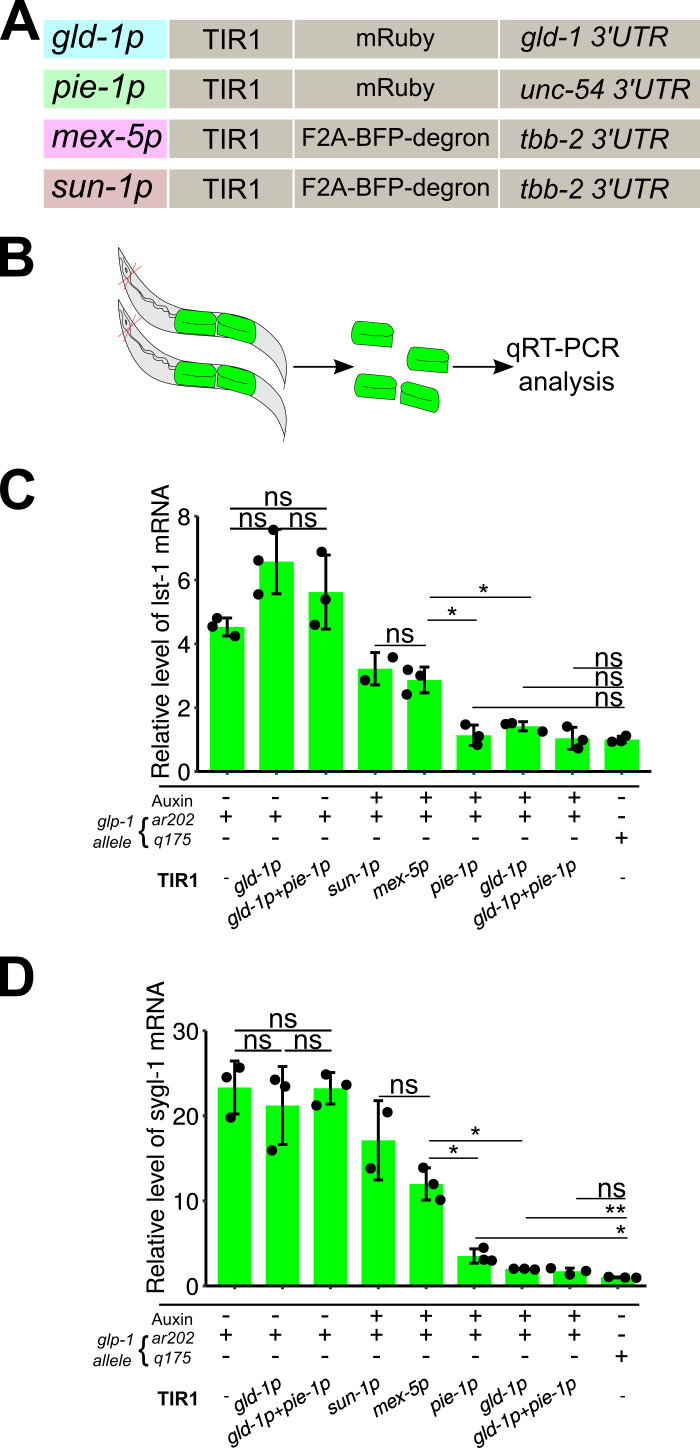
(A) Schematic showing four distinct TIR1 transgenes driven by different promoters, indicated by the gene name followed by “p”, for promoter. mRuby and BFP are fluorescent proteins, F2A is a self-cleavable linker. Not shown is the substrate, LAG-1::degron. (B) Schematic illustrating sample collection for qRT-PCR analysis. The animals were dissected and the extruded gonads collected for RNA purification and then qRT-PCR analysis. (C & D) The relative mRNA level of LAG-1 dependent transcriptional targets *lst-1* (C) and *sygl-1* (D) from dissected gonad samples of the indicated genotype and treatment. *q175* is a *glp-1* null allele, where the mRNA level of *sygl-1* and *lst-1* were normalized by setting their levels to one for this genotype. *ar202* is a *glp-1* gain of function allele. Results are from three biological replicates, except for *sun-1p::TIR1* (two replicates). Bar shows mean and standard deviation. Each dot represents a single data point. ns for not significant, * for p<0.01, and ** for p<0.001 by two-tailed *t-test*.

## Description

The *a*uxin *i*nducible *d*egradation (AID) system was first introduced in *C. elegans* by Dernburg lab and has become a widely-used approach to study tissue-specific and/or temporal aspects of gene function (Zhang *et al.* 2015; Ashley *et al.* 2020; Martinez *et al.* 2020). The AID system utilized a plant derived E3 ubiquitin ligase, TIR1, to specifically degrade proteins fused with the “degron” tag following treatment with the plant growth hormone auxin. To increase the utility of the AID system, the Ward laboratory recently generated an expanded set of TIR1s transgenes, controlled by different tissue-specific promoters (Ashley *et al.* 2020). Here we aim to compare different germline-expressed TIR1 transgenes for their efficiency in degrading the transcription factor LAG-1, which is C-terminally tagged with degron (Chen *et al.*, 2020). As indicated in Figure 1, these TIR1 transgenes are driven by the following promoters: *gld-1p* (Zhang *et al.* 2015), *mex-5p* (Ashley *et al.* 2020), *sun-1p* (Ashley *et al.* 2020), and *pie-1p* (Kasimatis *et al.* 2018), and contain the indicated C-terminal fluorescent proteins and 3’ untranslated regions (Fig. 1A).

In the *C. elegans* germline, GLP-1 Notch signaling employs transcriptional targets, *lst-1* and *sygl-1,* to maintain the germline stem cell (GSC) fate (Kershner *et al.* 2014; Chen *et al.* 2020). In the nucleus, the transcription factor LAG-1 and GLP-1(INTRA) form a complex that activates transcription of targets (Lee *et al.*, 2016; Chen *et al.*, 2020). LAG-1 is widely expressed and is also required for LIN-12 Notch signaling in somatic cells (Greenwald and Kovall 2013). Utilizing the AID system, we recently demonstrated that LAG-1 functions germline-autonomously to promote the GSC fate (Chen *et al.* 2020).

We have previously shown that the LAG-1 protein levels are reduced to background using the *gld-1p::TIR1* transgene (Chen *et al.* 2020). However, the low level of endogenous LAG-1 in the germline stem cell region, with or without auxin treatment, makes quantitative protein level comparisons challenging. Here, we measured the level of mRNA for the LAG-1 transcriptional targets *lst-1* and *sygl-1* by quantitative real-time PCR (qRT-PCR) from dissected gonads (Fig. 1B), as an indirect but more sensitive output for measuring degradation efficiency of LAG-1::degron by different TIR1 transgenes. Since LAG-1 degradation in the wild type germline caused the GSCs to enter meiosis, the qRT-PCR experiments were conducted in the *gld-2 gld-1* double mutant background (see complete genotype in the Reagent section). GLD-1 and GLD-2 are the two major pathways that promote GSCs to enter meiosis. In *gld-2 gld-1* double mutants the mitotically cycling germ cells fail to enter meiosis and results in a germline tumor, irrespective of GLP-1 signaling or LAG-1 function (Kershner *et al.* 2014; Chen *et al.* 2020). In addition, the *glp-1* gain of function allele *ar202* was included in the *gld-2 gld-1* double mutant background, allowing the transcription of *lst-1* and *sygl-1* mRNA to occur throughout the germline, thus increasing signal-to-noise ratio. The auxin treatment and qRT-PCR experiments were conducted as previously described (Chen *et al.* 2020). Starvation synchronized L1 animals were grown on NGM plates supplemented with or without 1 mM auxin for 48 hours at 25 °C. After auxin treatment, ~50 adult animals were dissected to isolate the gonads (Fig. 1B, Chen *et al.* 2020). The relative mRNA levels of *lst-1* and *sygl-1* are presented in Figure 1C & D.

One potential issue for the AID system is auxin independent TIR1 degradation of the target proteins. With the *lag-1::degron* allele, we have previously shown that both LAG-1 protein accumulation and progenitor zone size are largely normal in the presence of *gld-1p::TIR1*, without auxin (Chen *et al.* 2020). Here we compared the mRNA levels of *lst-1* and *sygl-1* from three different strains grown on NGM plates without auxin (Fig. 1C & D). The data showed that both *lst-1* and *sygl-1* mRNA level were not significantly different among animals without auxin regardless of which TIR transgene they carried (Fig. 1C & D), indicating that auxin independent TIR1 degradation of LAG-1 did not occur. We found that all four TIR1s can degrade LAG-1 sufficiently to generate Glp-1 like sterile animals in otherwise wild type background. In the tumorous germlines, the levels of *lst-1* and *sygl-1* mRNA were reduced in auxin treated animals, also supporting the conclusion that the AID system is knocking down LAG-1 protein. Importantly, we observed that *lst-1* and *sygl-1* mRNA level was lower in the *gld-1p::TIR1* and *pie-1p::TIR1* backgrounds compared to that in the *sun-1p::TIR1* and *mex-5p::TIR1* backgrounds, after auxin treatment (Fig. 1). When compared to *glp-1(q175)* null background, we noticed that the average expression level of *lst-1* and *sygl-1* mRNA was slightly higher in *gld-1p::TIR1,* *pie-1p::TIR1*, and *gld-1p::TIR1 +* *pie-1p::TIR1* background after auxin treatment. Statistical analysis indicates the *lst-1* mRNA level was indistinguishable compared to *glp-1* null germline, while the *sygl-1* mRNA level is slightly but significantly higher than that in *glp-1* null germlines. Together, we concluded that *gld-1p::TIR1* and *pie-1p::TIR1* are more efficient in degrading LAG-1 in the germline stem cell region.

It is not known what molecular feature(s) of the TIR1 transgenes determined high efficiency degradation of LAG-1::degron in germline stem cells, as the transgenes differ not only in the promoter, but also in the fluorescent protein tag, and in the 3’ UTR. For example, the *mex-5p* and *sun-1p* transgenes contain fluorescent protein BFP with a C-terminal degron tag, separated by a self-cleavable linker F2A, a design that allows a direct functional check of TIR1 activity through loss of BFP fluorescence. In contrast, *pie-1p::TIR1* and *gld-1p::TIR1* have mRuby fused to TIR1 without the degron motif. It is possible that reduced efficiency of *mex-5p* and *sun-1p* transgenes, rather than a consequence of different promoters, was due to competition of TIR1 for BFP::degron versus LAG-1::degron. Finally, we recommend testing multiple TIR1 transgenes to determine which one is optimal for the protein, cell type and timing of interest.

## Reagents

The following strains are used in this study:

BS3879:

gld-2(q497) gld-1(q485)/hT2::gfp [bli-4(e937) let-?(q782) qIs48] (I);

glp-1(q175)/hT2::gfp [bli-4(e937) let-?(q782) qIs48] (III)

BS4310:

gld-2(q497) gld-1(q485)/hT2::gfp [bli-4(e937) let-?(q782) qIs48] (I);

glp-1(ar202)/hT2::gfp [bli-4(e937) let-?(q782) qIs48] (III)

BS5629:

gld-2(q497) gld-1(q485)/hT2::gfp [bli-4(e937) let-?(q782) qIs48] (I);

ieSi64 [gld-1p::TIR1::mRuby::gld-1 3’UTR + Cbr-unc-119(+)] (II);

glp-1(ar202)/hT2::gfp [bli-4(e937) let-?(q782) qIs48] (III);

lag-1(oz536oz537[lag-1::degron::3xHA]) (IV)

BS5631:

fsIs1 [pie-1p::TIR1::mRuby::unc-54 3’UTR] gld-2(q497) gld-1(q485)/hT2::gfp [bli-4(e937) let-?(q782) qIs48] (I);

ieSi64 [gld-1p::TIR1::mRuby::gld-1 3’UTR + Cbr-unc-119(+)] (II);

glp-1(ar202)/hT2::gfp [bli-4(e937) let-?(q782) qIs48] (III);

lag-1(oz536oz537[lag-1::degron::3xHA]) (IV)

BS5630:

fsIs1 [pie-1p::TIR1::mRuby::unc-54 3’UTR] gld-2(q497) gld-1(q485)/hT2::gfp [bli-4(e937) let-?(q782) qIs48] (I);

glp-1(ar202)/hT2::gfp [bli-4(e937) let-?(q782) qIs48] (III);

lag-1(oz536oz537[lag-1::degron::3xHA]) (IV)

BS7023:

gld-2(q497) gld-1(q485)/hT2::gfp [bli-4(e937) let-?(q782) qIs48] (I);

wrdSi8 [mex-5p::TIR1::F2A::mTagBFP2::degron-NLS::tbb-2 3’UTR] (II);

glp-1(ar202)/hT2::gfp [bli-4(e937) let-?(q782) qIs48] (III);

lag-1(oz536oz537[lag-1::degron::3xHA]) (IV)

BS7024:

gld-2(q497) gld-1(q485)/hT2::gfp [bli-4(e937) let-?(q782) qIs48] (I);

cpIs103 [sun-1p::TIR1::F2A::mTagBFP2::degron-NLS::tbb-2 3’UTR] (II);

glp-1(ar202)/hT2::gfp [bli-4(e937) let-?(q782) qIs48] (III);

lag-1(oz536oz537[lag-1::degron::3xHA]) (IV)
